# Influence of PZT Coating Thickness and Electrical Pole Alignment on Microresonator Properties

**DOI:** 10.3390/s16111893

**Published:** 2016-11-10

**Authors:** Giedrius Janusas, Sigita Ponelyte, Alfredas Brunius, Asta Guobiene, Andrius Vilkauskas, Arvydas Palevicius

**Affiliations:** 1 Faculty of Mechanical Engineering and Design, Kaunas University of Technology, Studentu str. 56, Kaunas LT-51424, Lithuania; giedrius.janusas@ktu.lt (G.J.); alfredas.brunius@ktu.edu (A.B.); andrius.vilkauskas@ktu.lt (A.V.); arvydas.palevicius@ktu.lt (A.P.); 2 Institute of Materials Science, Kaunas University of Technology, Barsausko str. 59, Kaunas LT-51423, Lithuania; asta.guobiene@ktu.lt

**Keywords:** microresonator, PZT, poling, coating

## Abstract

With increasing technical requirements in the design of microresonators, the development of new techniques for lightweight, simple, and inexpensive components becomes relevant. Lead zirconate titanate (PZT) is a powerful tool in the formation of these components, allowing a self-actuation or self-sensing capability. Different fabrication methods lead to the variation of the properties of the device itself. This research paper covers the fabrication of a novel PZT film and the investigations of its chemical, surface, and dynamic properties when film thickness is varied. A screen-printing technique was used for the formation of smooth films of 60 µm, 68 µm, and 25 µm thickness. A custom-made poling technique was applied to enhance the piezoelectric properties of the designed films. However, poling did not change any compositional or surface characteristics of the films; changes were only seen in the electrical ones. The results showed that a thinner poled PZT film having a chemical composition with the highest amount of copper and zirconium led to better electrical characteristics (generated voltage of 3.5 mV).

## 1. Introduction

Today, piezoelectric materials are a very attractive method for the design of various micro-electro-mechanical systems (MEMS) and micro-opto-electro-mechanical systems (MOEMS). The importance of the piezoelectric effect in the micro dimension is imperative: it enables sensing and actuation performance, transforms strain into an electric current (and vice versa), and converts an electric field into strain [1,2]. 

Microresonators are devices where a piezoelectric coating plays the most important role in the design and operation of the element itself. In recent years, scientific researchers have concentrated on the fabrication and improvement of microresonators in different levels of components: design, electronic, mechanical, and control. A number of different types of microresonators are designed for different purposes: ceramic [3,4], acoustic [5,6,7], optical [8], pressure sensors [9], etc. Up-to-date technologies allow the attainment of a precise and sensitive design with a good signal-to-noise ratio (SNR) [10], a wide frequency range, a simplified digital interface, etc. However, the basic problem of these novel microresonators is the variation of their cross-section, leading to a complicated assessment of the resonant frequency and their high sensitivity to surface processes (e.g., a large surface area-to-mass ratio). The usage of piezoelectric materials in the design of microresonators requires a low driving voltage and enables self-sensing and self-actuation. It may be designed into a compact structure and easily integrated in any electronic circuit.

In this study, working with a cantilever-type structure allows the characterization of the properties of piezoelectric material coatings correctly—i.e., by analyzing resonance frequency shifts and dynamic responses. Characterization at low frequencies is more effective because of the appearance of large amplitude oscillation and due to potential nonlinear behavior.

Different methods of fabricating piezoelectric materials [11,12,13] lead to the variation of the properties of the device itself. This research article has utilized novel piezoelectric films integrated in the design of a microresonator. The elements designed in this study consist of copper foil, a layer of novel piezoelectric material (a sol-gel lead zirconate titanate, PZT), and a segment top of a copper electrode layer. Six kinds of cantilever-type microresonators were designed, each with a different thickness of a piezoelectric coating; three of them were poled, and the rest were not. However, poling did not make any change to surface and chemical composition, except for the piezoelectric properties. Therefore, the main data in this paper are given for poled elements, except the results of a dynamic response. The influence of the variation of piezoelectric coating thickness on the properties of a microresonator was examined. The selection of the correct geometrical configuration of the device enables researchers to design a microresonator working at a defined frequency range and having a high Q factor. The variation of the thickness of a piezoelectric coating allows parameters to be controlled according to the designed system requirements. This technique of cross-section variation is one of the effective methods to determine microresonators’ fundamental resonant frequency. The main advantages of the novel designed resonators are: a simple and compact structure, a low driving voltage, and low-cost fabrication process and materials.

## 2. Materials and Methods 

### 2.1. Synthesis and Formation of PZT Coating

An oxalic acid/water-based synthesis of nano powders of lead zirconate titanate [Pb (Zr_x_, Ti_1–x_) O_3_] with x = 0.52—also known as PZT (52/48)—was used. The precursors of PZT (52/48) solution were lead (II) acetate [Pb(NO_3_)_2_], titanium butoxide [Ti(C_4_H_9_O)_4_], and zirconium butoxide [Zr(OC_4_H_9_)_4_]. The other reagents used were oxalic acid, deionized water, acetic acid, and ammonia solution. Lead (II) acetate [Pb(NO_3_)_2_] (8.26 g) was poured into 100 mL of water. Then, acetic acid was added, and the solution was heated to 50 °C and mixed to dissolve. Thirty-two grams of oxalic acid was dissolved in 500 mL of water, then stirred with the titanium butoxide (5.1 g) and zirconium butoxide (7.65 g) at a concentration of 80%. Afterwards, the lead acetate solution was added to the titanium butoxide and zirconium butoxide solution. The final solution was alkalized with 25% ammonia solution to pH 9–10 and mixed for an hour. The precipitate of the solution was filtered in vacuum, and was washed with water and acetone during filtering. After filtering, the material was dried at 100 °C for 12 h. The powder was heated at 1000 °C for 9 h. Finally, PZT powder was milled and mixed with 20% solution of polyvinyl butyral in benzyl alcohol mixed under defined conditions: 80% of PZT and 20% of binding material. Finally, the paste was coated on a copper foil using a screen printing technique.

Three different types of polyester monofilament screen mesh were used in this research paper: 32/70, 48/70, 140/34. The coating was then dried in the furnace for 30 min at 100 °C. Different size screen mesh was chosen to control the thickness of the PZT coating. Thus, three coatings of different thickness were formed and investigated: element 1 with a PZT coating of 68 µm thickness, element 2 with 60 µm thickness, and element 3 with 25 µm thickness (see Table 1). 

### 2.2. Analytical Methods for the Evaluation of PZT Coating Properties

#### 2.2.1. Electrical Pole Alignment of the Coating 

**Figure 1:**
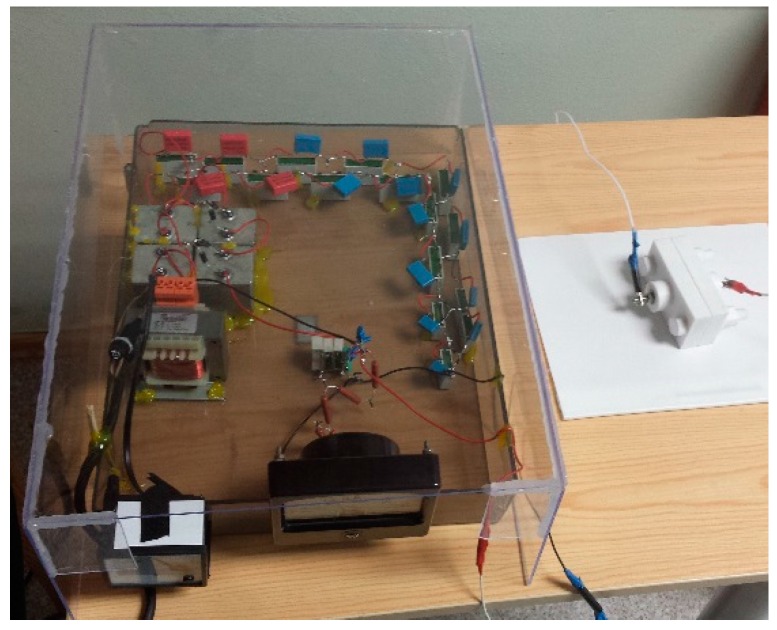
Pole alignment set.

Before measuring the generated voltage, an electrical pole alignment was applied on a PZT coating of the microresonators. It was accomplished with a high voltage generator and a custom-made holder, shown in Figure 1. An element with a PZT coating was placed in the special holder between positive and negative poles. The high voltage generator was set at 5 kV current and held for 30 min. The poling technique aligns a positive pole on one side of the PZT coating and a negative pole on the other side. This process improves voltage characteristics of a piezoelectric coating.

#### 2.2.2. Structural and Chemical Composition Measurements

The structure and chemical composition of the designed material was investigated using scanning electron microscope (SEM) Quanta 200 FEG, also integrated with the energy dispersive X-ray spectrometer (EDS) detector X-Flash 4030 from Bruker. Samples were examined under the atmosphere of a water steam of controlled pressure. A 133 eV (at Mn K) energy resolution at 100,000 cps was achieved with a 30 mm^2^ area solid state drift detector, cooled with a Peltier element. The X-ray spectroscopy method allows for the analysis of energy distributions. The energy differences were measured between various quantum states of the system, together with the probabilities that the system jumps between these states.

Fourier transform infrared spectroscopy (FTIR, SPECTRUM GX 2000 RAMAN, PerkinElmer, Waltham, MA, USA) was used for the investigation of changes in chemical composition when the coating was poled and not poled. The diapason of FTIR spectrum was 10,000–200 cm^–1^. This technique enabled the researchers to identify changes in chemical compounds of the elements. 

For qualitative and quantitative analysis of chemical compounds, an X-ray diffractometer D8 Discover (Bruker, Billerica, MA, USA) was used. The atomic and molecular structure of the designed PZT was identified.

#### 2.2.3. Evaluation of Surface Morphology

The investigations of surface morphology were performed with an atomic force microscope NT-206 in contact mode. Atomic force microscopy is a surface analytical technique used to generate very high-resolution topographic images of surfaces down to molecular/atomic resolution, the sample being deposited on a flat surface being the only requirement. Depending on the sharpness of the tip, it gives spatial resolutions of 1–20 nm. It can record topographic images in addition to providing some information on nanoscale chemical, mechanical (modulus, stiffness, viscoelastic, frictional), electrical, and magnetic properties when using specialized modes. Morphology parameters are as follows: *Z_mean_*, average height; *R_a_*, arithmetic average surface roughness; *R_q_*, root mean squared surface roughness.

#### 2.2.4. Dynamic Investigations of PZT Coatings 

**Figure 2:**
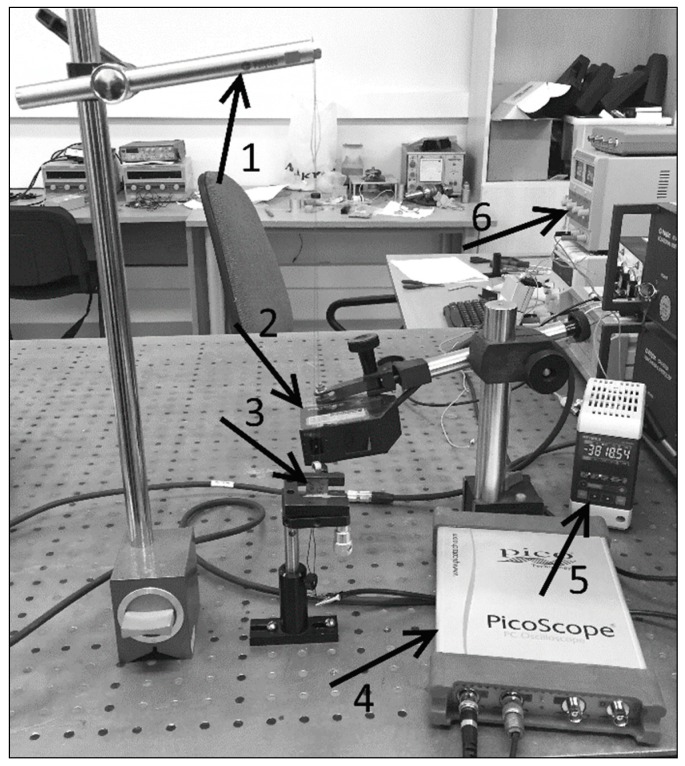
Experimental setup consisting of: (**1**) a mathematical pendulum; (**2**) a LK-G82 sensor head; (**3**) an investigated element; (**4**) PicoScope oscilloscope; (**5**) LK-G3001PV control block; and (**6**) a power supply block.

The experimental setup of this investigation consisted of a piezoelectric energy harvester (PVEH) applying single hits, excitation, and measurement systems and data acquisition. The data acquisition system consisted of a four-channel USB oscilloscope (analog-to-digital converter) PicoScope 6000 series (Pico, Cambridgeshire, UK) (Figure 2) that collects signals from the accelerometer and PVEH. Signals from the oscilloscope are forwarded to the computer and managed with PicoScope 6000 software. The system is based on a mathematical pendulum (Figure 2). 

The experimental system was designed as a mathematical pendulum, which provides a single impulse to the clamped element when indicated. The response of vibrations was sensed with an accuracy of 0.2 μm using a LK-G3000 series laser triangular displacement sensor (sensor head LK-G82, control block LK-G3001PV) (Keyence, IL, USA), and the measured data was collected with a PicoScope data acquisition system (with data reading velocity 5 Gs/s).

## 3. Results

### 3.1. Structure and Chemical Composition 

A dispersive X-ray spectrometer was used here for energy distribution analysis.

**Figure 3:**
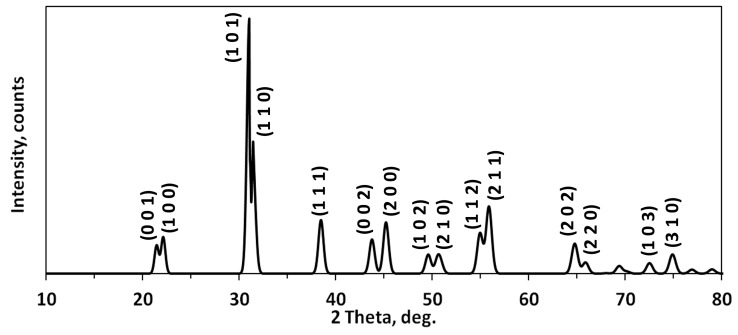
X-ray diffraction (XRD) pattern of lead zirconate titanate (PZT) powder after the final calcination process.

Figure 3 shows the XRD pattern of PZT powder after the final calcination process. PZT ceramics crystallize in a tetragonal structure (a = b = 4.006 Å, c = 4.128 Å, α = β = γ = 90 deg.) with space group P 4 mm (noncentrosymmetric) and the (0 0 1), (1 0 0), (1 0 1), (1 1 0), (1 1 1), (0 0 2), (2 0 0), (1 0 2), (2 1 0), (1 1 2), (2 1 1), (2 0 2), (2 2 0), (1 0 3), and (3 2 0) crystallographic plane orientations, corresponding to values reported in [14,15]. The XRD pattern of PZT powder corresponds to Pb(Zr_0.52_Ti_0.48_)O_3_ with R_f_ factor of 0.31. 

**Figure 4:**
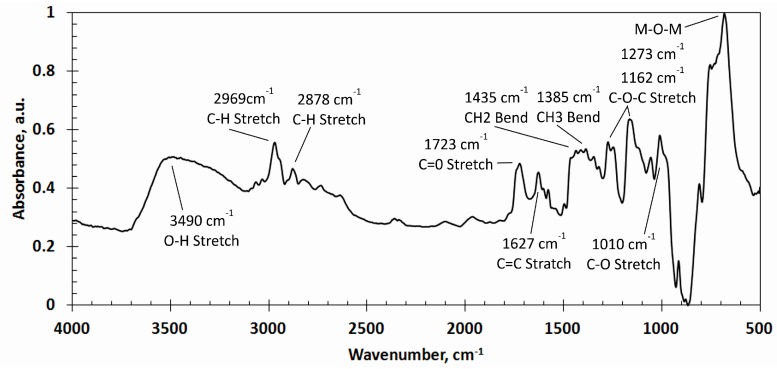
Fourier transform infrared spectroscopy (FTIR) absorbance spectra with functional groups of PZT coating.

Fourier transform infrared spectroscopy FTIR analysis of a non-poled and poled PZT coating applied using different screen-printing meshes was carried out. There was no significant influence of poling and thickness upon the spectrum of PZT; therefore, the typical FTIR absorbance spectrum at 4000–500 cm^–1^ of the PZT coating is presented in Figure 4. 

In the FTIR spectra, strong and broad absorption peaks were observed at 3490 cm^–1^ (O–H stretch), 2969 cm^–1^ (C–H stretch), 2878 cm^–1^ (C–H stretch), 1723 cm^–1^ (C=O stretch), 1627 cm^–1^ (C=C stretch), 1435 cm^–1^ (CH2 bend), 1385 cm^–1^ (CH3 bend), 1273 cm^–1^ (C–O–C stretch), 1162 cm^–1^ (C–O–C stretch), and 1010 cm^–1^ (C–O stretch). The entire array of these peaks corresponds to the FTIR absorbance spectra of polyvinyl butyral (PVB) [15]. A wide and strong peak observed in the range of 800–550 cm^–1^ corresponds to the M–O–M bonds (M is metal) of PZT (e.g., Ti–O, Ti–O–Ti, Zr–O, and Zr–O–Zr) [16].

**Figure 5:**
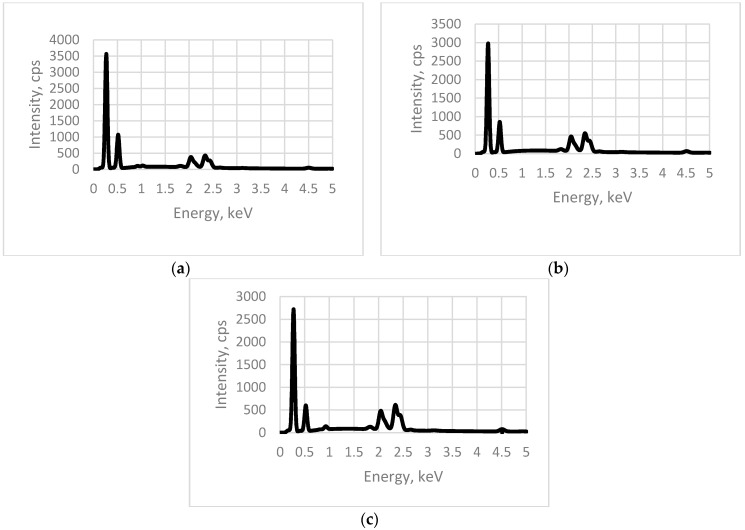
Energy dispersive spectrum of: (**a**) Element 1 (mesh 32/70); (**b**) Element 2 (mesh 48/70); (**c**) Element 3 (mesh 140/34).

The structure and chemical composition of the designed PZT material was investigated using a Quanta 200 FEG scanning electron microscope (Hillsboro, OR, USA) integrated with the X-Flash 4030 energy dispersive X-ray spectrometer detector from Bruker (Berlin, Germany). Three elements, 1, 2, and 3 (poled, with different thicknesses) were examined under the atmosphere of a water steam of controlled pressure. A 133 eV (Mn·Kα) energy resolution at 100,000 cps was achieved with a 30 mm^2^ area solid state drift detector, cooled with a Peltier element. The energy differences were measured between various quantum states of the system together with the probabilities that the system jumps between these states. The obtained results clearly show that PZT is dominant in the composition of all three elements (Figure 5).

The comparison of EDS samples with different layers shows that there are no significant differences in the locations of peaks. Comparing EDS patterns of layers of different thickness, it is observed that the intensities of peaks for PZT elements (Pb, Ti, Zr) increased for a thinner layer; i.e., a thinner layer has a greater concentration of PZT, which provides better piezoelectric properties.

**Figure 6:**
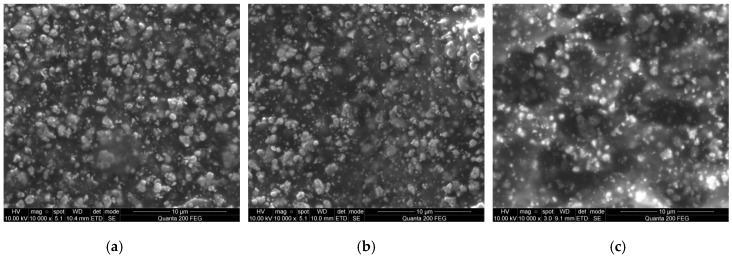
SEM views of samples: (**a**) Element 1 (mesh 32/70); (**b**) Element 2 (mesh 48/70); (**c**) Element 3 (mesh 140/34).

SEM micrographs of the investigated elements are given in Figure 6. Results show that element 1 has small granular grains on the surface of a diameter ~1.1 µm. Element 2 has a smoother surface, with fewer grains of a diameter ~0.9 µm. Element 3 has three-dimensional structures with empty cavities of a 6–8 µm diameter.

**Figure 7:**
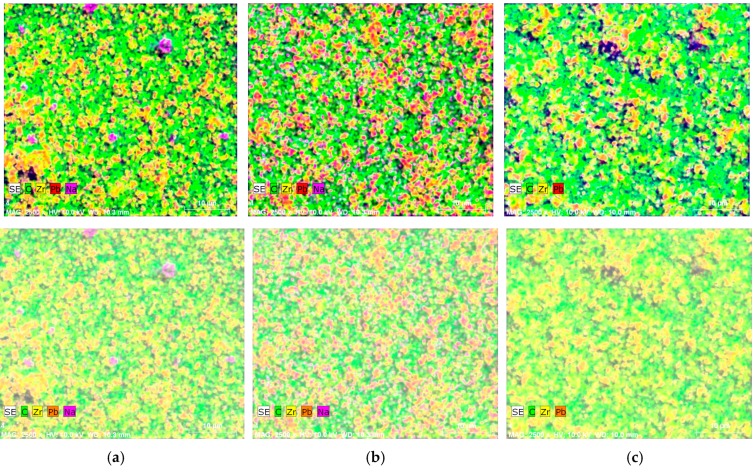
Elemental mapping done with SEM of the (**a**) Element 1 (mesh 32/70); (**b**) Element 2 (mesh 48/70); (**c**) Element 3 (mesh 140/34).

Full composition of the elements was defined in Figure 7. The main elements in the composition are Carbon (C) and Zirconium (Zr); both are very good conductors defining good piezoelectric properties of designed novel coatings. 

### 3.2. Surface Morphology 

**Figure 8:**
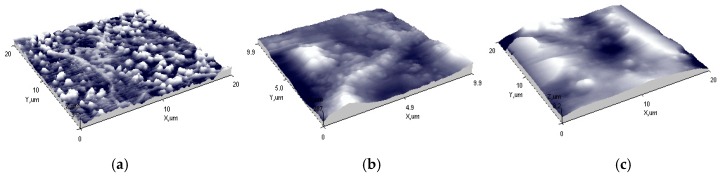
Atomic force microscopy (AFM) 3D view of: (**a**) Element 1 (mesh 32/70); (**b**) Element 2 (mesh 48/70); (**c**) Element 3 (mesh 140/34).

Atomic force microscopy was used to evaluate the surface morphology of the designed elements. 3D views show that element 1 has a rather smooth surface, with roughness R_q_ = 29 nm (Figure 8a). Elements 2 and 3 have rough surfaces with roughness R_q_ = 189 nm and R_q_ = 149 nm, respectively (Figure 8b,c). Using a different screen-printing mesh allows controlling not only thickness but also surface morphology of the element (Table 2). 

### 3.3. Energy Harvesting of the Elements

**Figure 9:**
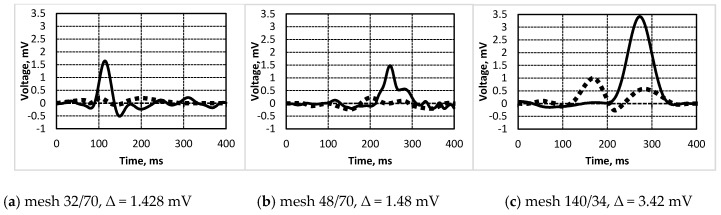
Generated voltage diagram of (**a**) Element 1 (―poled, ▪▪▪ not poled); (**b**) Element 2 (―poled, ▪▪▪ not poled); (**c**) Element 3 (―poled, ▪▪▪ not poled).

Elements (poled and not poled) were investigated with PVEH based on direct and indirect piezoelectric effects. Results showed that no signals were received when indirect piezoelectric was applied; i.e., there were no significant vibrations under various frequencies and different bias for both poled and not poled elements. However, the investigations based on the direct piezoelectric effect showed remarkable results; i.e., under impulse force of 5 N amplitude applied on the poled element, it generated from ~1.4 mV to ~3.5 mV (Figure 9). Poled element 3—the one with the thinnest PZT layer—generated the highest voltage of 3.6 mV (Figure 9c). It was easy to detect the difference between poled and not poled elements—i.e., around 61% less of a generated voltage.

During poling, the material is subjected to a very high electric field that orients all the dipoles in the direction of the field. Upon switching off the electric field, most dipoles do not return to their original orientation as a result of the pinning effect produced by microscopic defects in the crystalline lattice. This gives a material comprising numerous microscopic dipoles that are roughly oriented in the same direction. 

The novelty is in the designed material PZT; i.e., the obtained material is not classic PZT, with wider application areas. The designed microresonator may be operated in a system with an indicated resonant frequency by varying dimensions of the microresonator’s layers and its geometrical parameters. The aim of this research was to create a novel microresonator with controllable parameters that could assure much higher functionality of MEMS. Creation of this novel element will allow it to integrate in various MEMS systems: high stability electric oscillation sources (as generators), electric filters, in energy harvesting, sensors for testing proteins, viruses, chemical species, etc.

## 4. Conclusions

The main elements in the PZT film composition were Carbon (C) and Zirconium (Zr)—both are very good conductors defining good piezoelectric properties.

No significant differences in chemical composition and surface morphology were determined when elements were poled and not, except for the dynamic response. 

Results showed that under the impulse force of 5 N amplitude applied on the poled element, it generated from ~1.4 mV to ~3.5 mV; i.e., the thinner the PZT layer, the more power it generates when affected mechanically.

The poled element generated around 61% more power compared to the one which was not poled.

Using determined dimensions of the microresonator’s layers with its geometrical parameters allows the microresonator to be operated at a resonant frequency suitable for a particular application. 

## Figures and Tables

**Table 1:** Properties of screen mesh and layer thickness.

Meshed Screen Type	Mesh Opening, µm	Thread, µm	Open Area, %	Mesh Thickness, µm	Theoretical Ink Volume, cm^3^/m^2^	Formed PZT Layer Thickness, µm
32/70	245	70	60.5	108	65	68 ± 1
48/70	130	70	42.3	107	46	60 ± 1
140/34	30	34	22	52	11	25 ± 1

**Table 2:** AFM values of the surface morphology.

Element	Mesh	*Z_mean_*, nm	*R_a_*, nm	*R_q_*, nm
1	32/70	54	21 ± 1	29 ± 1
2	48/70	396	156 ± 0.5	189 ± 0.5
3	140/34	457	112 ± 0.5	149 ± 0.5
